# Supplementary material for: An intergenerational androgenic mechanism of female intrasexual competition in the cooperatively breeding meerkat
Source: Nat Commun. 2021 Dec 17;12:7332. doi: 10.1038/s41467-021-27496-x (PMC8683399; doi:10.1038/s41467-021-27496-x)
Supplement: Supplementary file 1 — Supplementary Information [file 41467_2021_27496_MOESM1_ESM.docx]

Supplementary Information

**An intergenerational androgenic mechanism of female intrasexual competition in the cooperatively breeding meerkat**

Christine M. Drea^1,2,3^*, Charli S. Davies^1,3,6^, Lydia K. Greene^1,3,7^, Jessica Mitchell^1,3,8^, Dimitri V. Blondel^1,3,9^, Caroline L. Shearer^1^, Joseph T. Feldblum^1,10^, Kristin A. Dimac-Stohl^1,2^, Kendra N. Smyth-Kabay^1,3,11^ & Tim H. Clutton-Brock^3,4,5^

^1^Department of Evolutionary Anthropology, Duke University, Durham, NC 27708 USA

^2^Department of Biology, Duke University, Durham, NC 27708 USA

^3^Kalahari Research Trust, Kuruman River Reserve, Northern Cape, South Africa

^4^Department of Zoology, University of Cambridge, Cambridge, CB2 3EJ UK

^5^Mammal Research Institute, University of Pretoria, 0002 Pretoria, South Africa

^6^School of Biological Sciences, University of East Anglia, Norwich Research Park, Norwich, NR4 7TJ UK

^7^Duke Lemur Center, Duke University, Durham, NC 27705 USA

^8^Nuffield Centre for International Health and Development, University of Leeds, Leeds, West Yorkshire, LS2 9JT UK

^9^Department of Biology, North Carolina Wesleyan College, Rocky Mount, NC 27804 USA

^10^Department of Anthropology and Society of Fellows, University of Michigan, Ann Arbor, MI 48109 USA

^11^Boston Consulting Group, Bethesda, MD 20814 USA

*Correspondence to: cdrea@duke.edu

**This PDF file includes:**

Supplementary Methods

Supplementary Figures 1-7

Supplementary Tables 1-7

**Supplementary Methods**

**Enzyme immunoassays.** We determined serum androgen concentrations using commercial, competitive enzyme immunoassay (EIA) kits (ALPCO diagnostics, Salem, NH, USA)^15^, with assay sensitivities reported in the main text. We validated the EIA serum assays by standard parallelism, linearity and recovery tests. Samples with concentrations greater than the upper detection limit were diluted with assay buffer to no more than 1:8, and the results obtained were then multiplied by the dilution factor. Samples that had concentrations below the minimum detectable limit of the assay were allocated this minimum value. All samples were run in duplicate and were re-run in a subsequent assay if the coefficients of variation (CV) exceeded 10%. Assay sensitivities are reported in the main text. Serial dilutions of pooled meerkat serum androstenedione (A_4_) yielded a displacement curve parallel to the A_4_ standard curve. Assay accuracy, measured as percent recovery of known amounts of analyte from a pooled serum sample was 100.3% (n = 6). Cross reactivity of the A_4_ assay was 1.8% with dehydroepiandrosterone (DHEA), 0.2% with testosterone (T), < 0.1% with estrone, E_2_, progesterone, 17-OH progesterone and 5α-dihydrotestosterone (DHT), < 0.01% with cortisol and DHEA sulphate (DHEA-S). Serial dilutions of pooled meerkat serum yielded a displacement curve parallel to the T standard. Accuracy was 110% (n = 6). Cross reactivity of the T assay was 5.2% with DHT, 1.4% with A_4_, 0.8% with androstanediol, 0.5% with progesterone, 0.1% with androsterone and < 0.1% with aldosterone, andrenosterone, cholesterol, corticosterone, DHEA, DHEA-S, epiandrosterone, E_2_, estriol and pregnenolone.

Our EIA protocol for fAM was as follows^15^: we initially coated 96-well microtiter plates with 100 µL of a 1:6000 dilution of the antibody in a carbonate-bicarbonate buffer (Thermo Scientific). We then allowed plates to gently shake at room temperature for 2 hours. Afterwards, we rinsed plates once with 300 µL of a wash buffer (90% de-ionized water; 10% phosphate-buffered saline (PBS); 0.03% bovine serum albumin (BSA); and 0.05% tween-20), and blocked plates at room temperature for one hour with 300 µL of blocking buffer (98% wash buffer and 2% BSA). While plates were blocking, we prepared a 1:1500 solution of the conjugate in wash buffer. We then dried faecal methanol extracts under a steady stream of nitrogen gas and resuspended (1:2.5) in the conjugate solution. Those samples with concentrations greater than the upper detection limit were re-run using a reduced volume of faecal methanol extract; the results were then multiplied by the dilution factor. When concentrations were below the range of detection, we reran those samples using a greater volume of faecal methanol extract (faecal extract: 1:2). T standards (12.5-0.2 ng/mL/well) were similarly prepared in the conjugate solution via serial dilution. We then pipetted into duplicate wells 100 µL of standards, blanks, and faecal samples and allowed plates to gently shake at room temperature for 1 hour. We washed the wells 3 times with 300 µL of wash buffer, and patted the wells dry. We pipetted 100 µL of 1-step Ultra TMB-ELISA Substrate (Thermo Scientific) to each well and allowed colour to develop for 12-15 min, until blank wells became dark blue. Lastly, we used 50 µL of a stop solution (2M sulphuric acid), and used a microplate reader from Biotek with the Gen5 software (version 1.10) to determine absorbance at 450 nm. Assay sensitivity is reported in the main text. Cross reactivity of the fAm assay was 100% with T, 9% with DHT, < 1% with androstenediol, and < 0.1% with A_4_, estriol, E_2_ and P_4_.

Faecal extracts for fGCm analysis were assayed using the ImmuChem double-antibody ^125^I radioimmunoassay kit for corticosterone (MP Biomedicals, Irvine, CA)^22^. Cross reactivity of the corticosterone assay was 0.34% with desoxycorticosterone, 0.10% with T, 0.05% with cortisol, 0.03% with aldosterone, 0.02% with P_4_, 0.01% with A_4_ and DHT, and < 0.01% with all other steroids tested. The minimum detectable dose was 7.7 ng/ml. Consistent with the validated protocol, we ran all volumes at one-half the recommended volumes for the kit. Assay accuracy, assessed by the recovery of standards added in duplicate to an extracted faecal sample, was 100.075% (±6.528 SD). Serial dilution of an extracted sample produced curves parallel to the standard curve.

**Behavioural categories and intra- and inter-observer reliability.** Following a previously established ethogram^43^, our aggression category includes both low-intensity behaviour, such as blocking an animal’s approach, and vocalisations, such as chatter and growling, as well as high-intensity aggression (HIA), such as food competition, bite, hip slam, push, social chin rub and threaten. Competition over an acquired food item is a frequent, key behaviour in meerkat society, so we also considered it separately from other HIA. Scent marking includes anal marking and overmarking, chew marking, frenzied body-rubbing or chin-rubbing of the environment, defecation and urination. Prosociality includes all forms of social play, as well as social grooming, social sniffing and huddling. Submission includes groveling and peep vocalisations. Vigilance includes guarding, raised guarding and scanning. Digging for food involves directed behaviour to unearth insects and, in this case, excludes surface scratching. Both vigilance and digging occur over appreciable periods of time and, thus, are recorded as both events and states.

To assess intra- and inter-observer reliability in obtaining our behavioural measures, we video recorded focal subjects behaving under conditions identical to those during our observation sessions, both at the den and whilst foraging. The various observers then scored these videos, in random order, on two separate occasions. For intra-observer reliability, we calculated indices of concordance for each observer and for each behavioural category. For inter-observer reliability, we compared the average scores across video trials for all observers, again calculating indices of concordance for each behavioural category.

**Supplementary Figures**


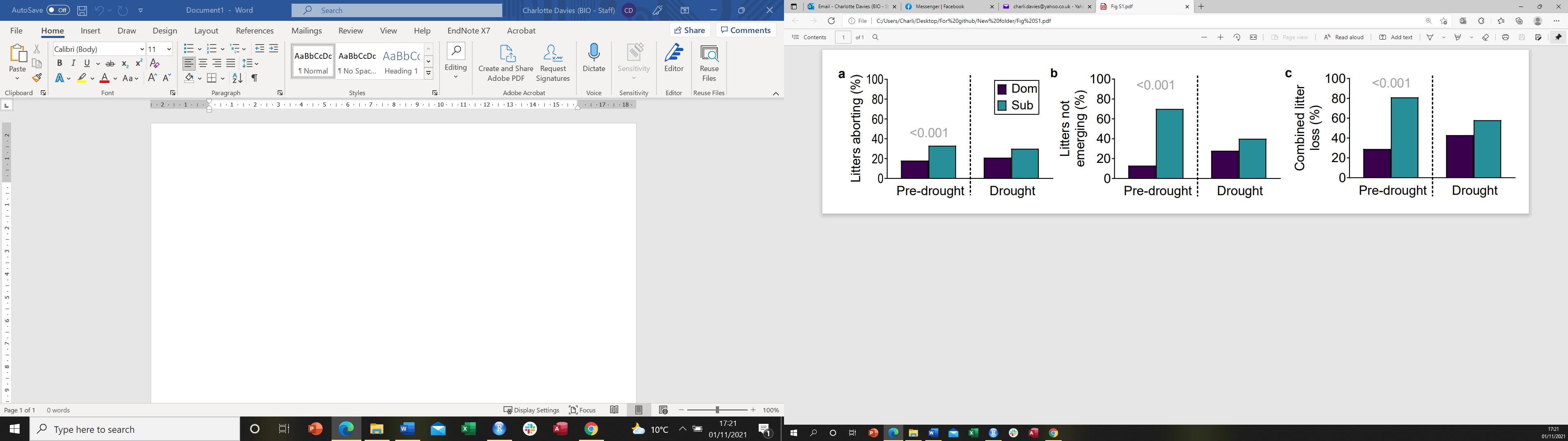
Supplementary Figure 1. Rainfall in the Kalahari impacts status-related reproductive loss in wild female meerkats. Rainfall during a pre-drought period (1994-2005)^21^ and during a drought (2011-2015)^22^ affects the abundance of insects (the primary food source of meerkats) and differentially impacts the percentages of a spontaneous abortions, b neonatal loss pre-emergence from the den and c both factors combined in dominant (Dom, purple) and subordinate (Sub, teal) females. Relative to the earlier time frame, drought conditions during the present study were associated with both a relative increase in the early reproductive loss of dominant females and a relative decrease in the early reproductive loss of subordinate females, which combined to minimize female reproductive skew. Analyses used Fisher's Exact test, two-tailed, based on 590 pregnancies pre-drought and 141 pregnancies during the drought; significant *P* values in grey. These figures were created from published data; see original publications^21,22^ for additional statistical comparisons.

**
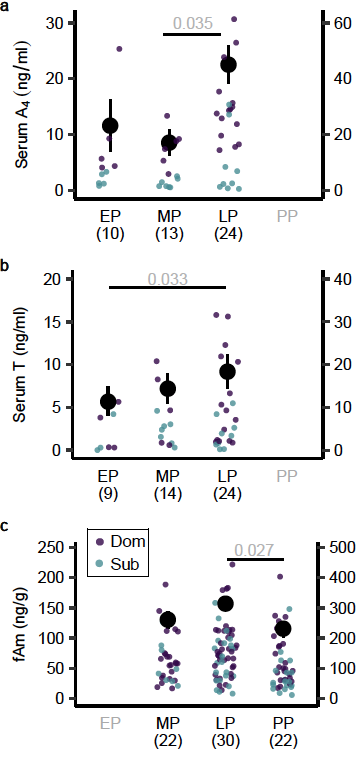
**

**Supplementary Figure 2. Normative distribution of androgen concentrations** **from early pregnancy to post-parturition in wild meerkats.** Concentrations of **a** serum androstenedione (A_4_), **b** serum testosterone (T) and **c** faecal androgen metabolites (fAm) change significantly across early pregnancy (EP), mid pregnancy (MP), late pregnancy (LP) and the early postpartum (PP) period in meerkats, with peak values occurring in LP (analyses are as described in Fig. 1a-c). Mean ± s.e. hourly rates (black) on the left y axis; raw data distribution (colour-coded by female status: dominant or Dom, purple; subordinate or Sub, teal) on the right y axis (to accommodate zero inflation). Values in parentheses represent the number of combined dominant and subordinate pregnancies; significant *P* values in grey. Source data are provided as a Source Data file.

**
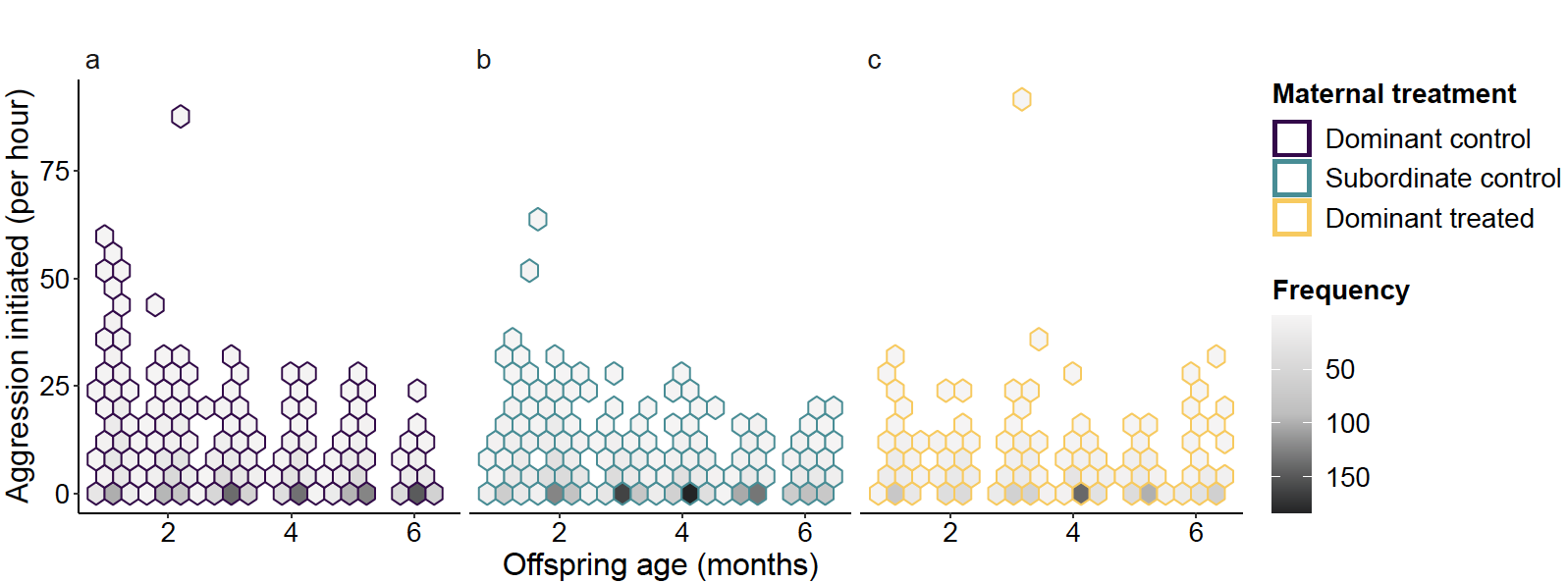
**

**Supplementary Figure 3. Hexagonal density plots of observed offspring aggression by age, subset by maternal status and treatment condition.** Offspring are from **a** dominant control (purple), **b** subordinate control (teal) and **c** dominant treated (orange) dams. Hexagons represent offspring aggression initiated (frequency per hour) during the first 6 months of life, with age-related frequencies represented by shading (higher frequency = darker grey; lower frequency = lighter grey). Data shown are truncated to exclude focals of short duration (< 5 min; 599 of 5661 or ~10.6% of focals were excluded) to minimize outliers; all data were included in the analysis (as described in Fig. 2). Source data are provided as a Source Data file.


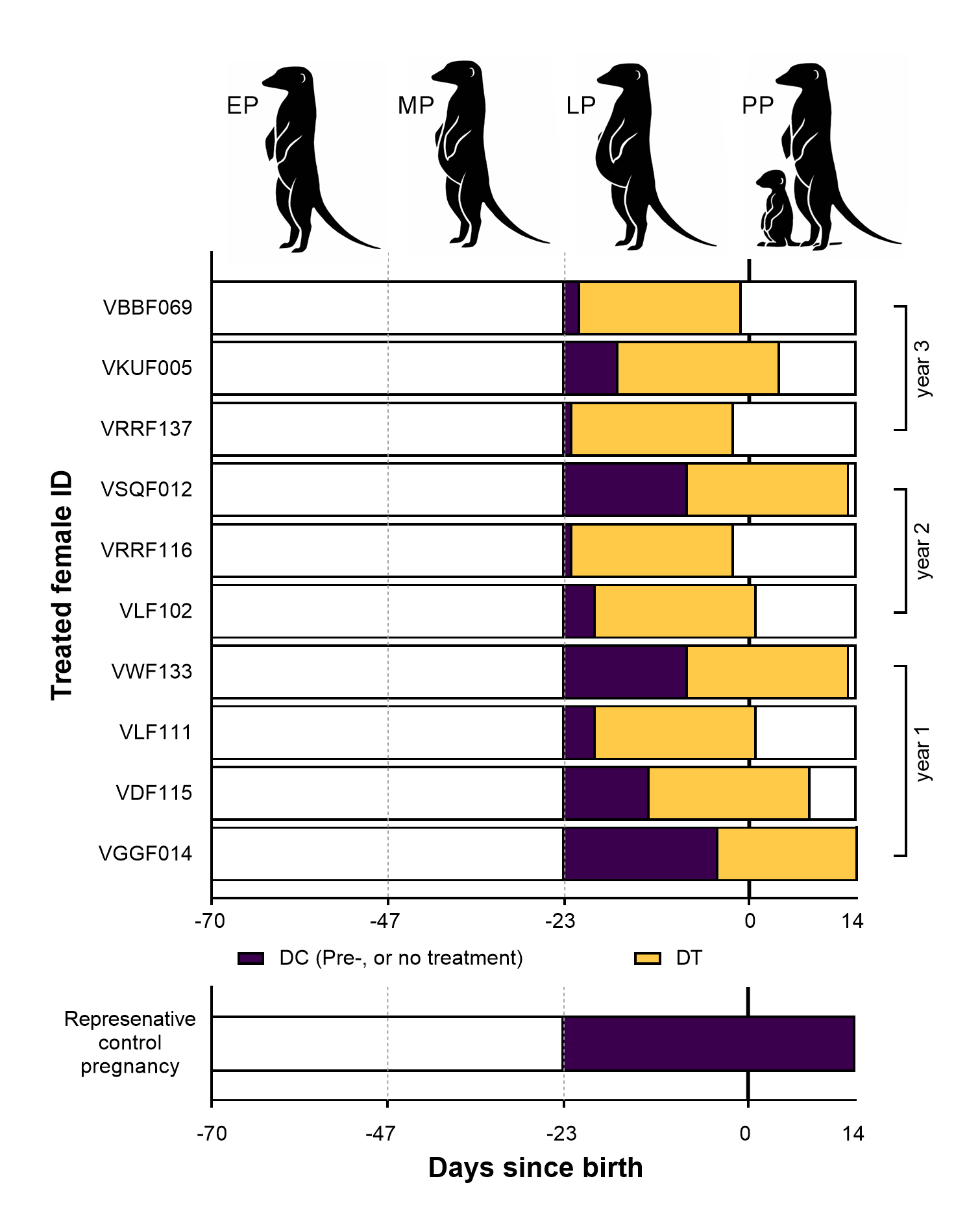


Supplementary Figure 4. Time course of late-pregnancy, antiandrogen treatment in individual dominant meerkats. Across three years of study, 10 dominant dams across 11 pregnancies received the androgen receptor blocker flutamide; 10 of those pregnancies (shown here) were carried to term. Treatment targeted the last 21 days of their 70-day gestation period, although with regard to developing infants, the critical period for differentiation of brain substrates underlying behaviour is expected to be shorter than that (i.e., on the order of days) and may extend postpartum. The dark, discontinuous vertical line at day 0 on the x axis represents parturition, dividing pregnancy (backdated from birth) from a two-week postpartum (PP) period. Stippled vertical lines further delineate early (EP), mid (MP) and late (LP) pregnancy. The achieved time courses of antiandrogen treatment (orange) in dominant treated (DT) dams are shown relative to each female’s own LP control period (purple). Also shown is a comparable control period extending into PP (purple) for a representative dominant control dam (DC) from a pool of 22 females and 31 pregnancies (see Table 2 in main text); EP, MP, and untreated portions of PP (white) were excluded from the analyses comparing dominant control dams to dominant treated dams. (Icons of pregnancy stages drawn by S. Bornbusch). Source data are provided as a Source Data file.


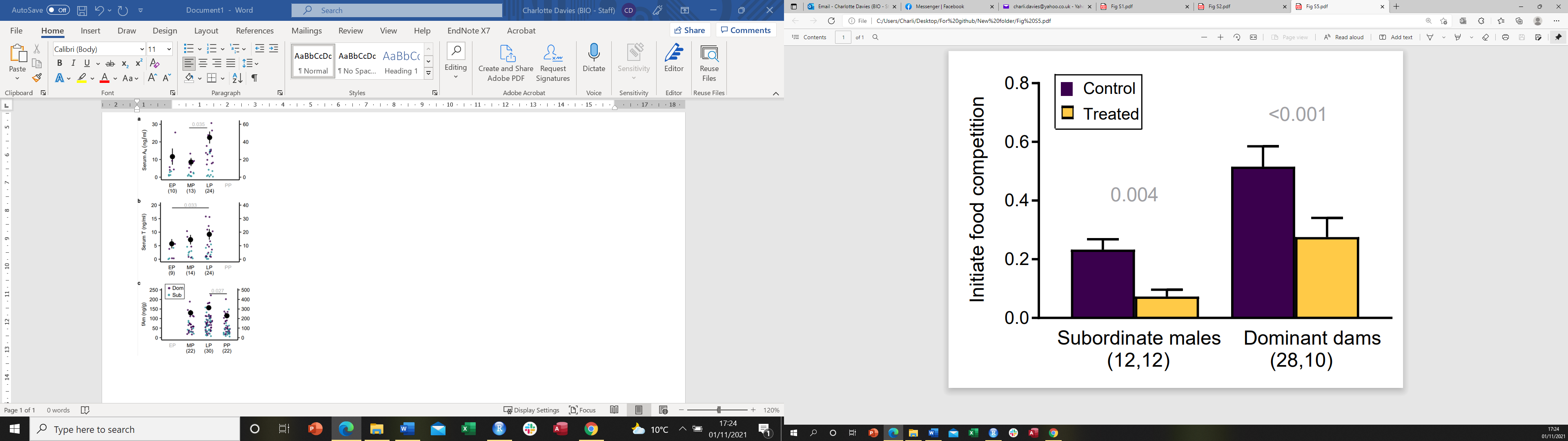


**Supplementary Figure 5. Consistent effects of antiandrogen treatment on the initiation of aggression in adult male and female meerkats.** Treatment with the antiandrogen flutamide reduces hourly rates of initiating food competition (comp) in adult subordinate males and dominant dams relative to control peers: control (purple); treated (yellow). Analyses used GLMMs, with two-tailed χ^2^_,_ based on 24 males observed during 524 focal sessions, and 31 pregnancies of 22 dominant dams observed during 799 focal sessions whilst ranging. Although females are more aggressive than are males, both sexes show proportionally equivalent reductions. Mean + s.e.: male values are extracted from published data^43^ and female values replicate those in Fig. 3a. Values in parentheses represent the numbers of males observed and the numbers of pregnancies; significant *P* values in grey. Source data are provided as a Source Data file.


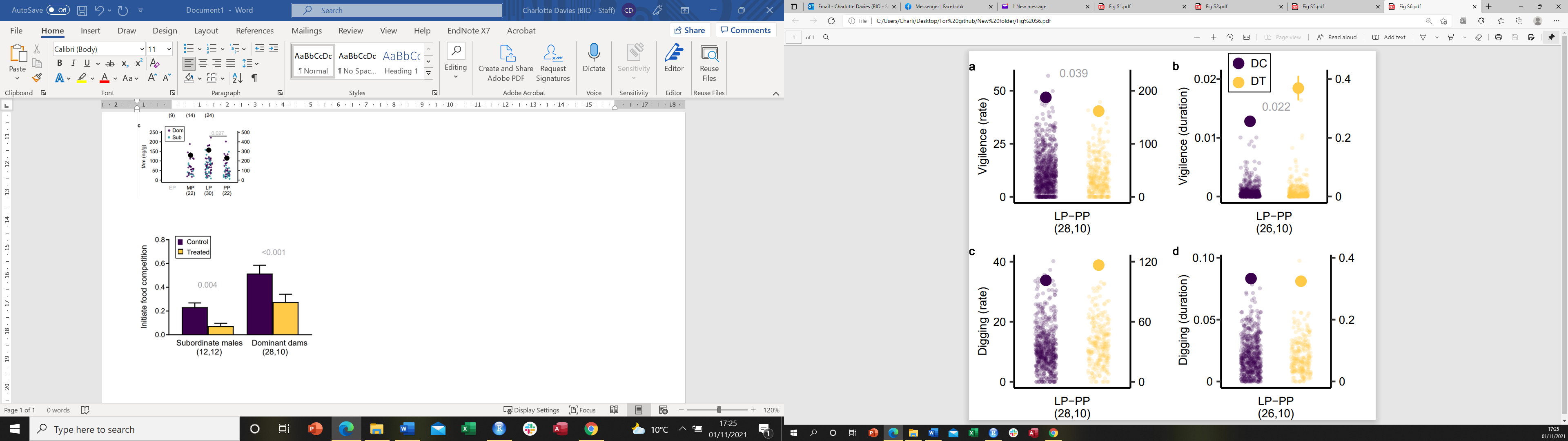


**Supplementary Figure 6. Lack of overall effects of concurrent antiandrogen treatment on neutral behaviour in pregnant dominant meerkats.** Treatment of dams across late pregnancy (LP) and early postpartum (PP) reduces **a** the rate (frequency per hour) of vigilance, but increases **b** the duration (in hours) of vigilance; neither **c** the rate of digging nor **d** the duration of digging showed any effect of treatment, either individually or overall. Dominant control (DC, purple); dominant treated (DT, orange). Mean ± s.e. on the left y axis; raw data on the right y axis (to accommodate zero inflation). Analyses used GLMMs for rates and LMMs for durations, based on 31 pregnancies of 22 dominant dams, observed during 1044 focals. Values in parentheses represent the number of pregnancies sampled per condition; significant *P* values in grey. Source data are provided as a Source Data file.


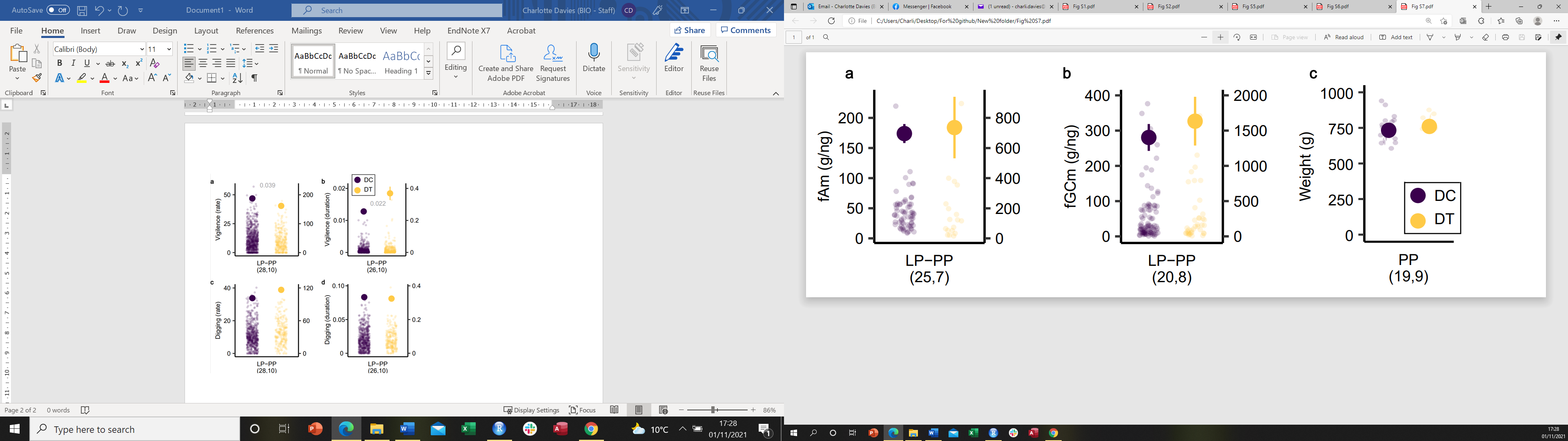


**Supplementary Figure 7. Lack of effects of concurrent antiandrogen treatment on faecal steroid concentrations and postpartum body weight in pregnant dominant meerkats.** Treatment of dams across late pregnancy (LP) and early postpartum (PP) had no effect on **a** concentrations of faecal androgen metabolites (fAm), **b** concentrations of faecal glucocorticoid metabolites (fGCm) or **c** body weight. Dominant control (DC, purple); dominant treated (DT, orange). Mean ± s.e. on the left y axis; raw data on the right y axis (to accommodate zero inflation). Analyses used LMM for endocrine data (83 faecal samples from 23-28 pregnancies) and LM for weights (28 pregnancies from 21 females). Values in parentheses represent the numbers of pregnancies sampled per condition. Source data are provided as a Source Data file.

| **Supplementary Table 1** **Androgen concentrations in pregnant meerkats in relation to status and pregnancy stage** | | | | |
| --- | --- | --- | --- | --- |
| **Androgen** | **Model terms** | **Estimate (SE)** | **χ^2^** | ***P*** |
| Androstenedione^a^ | **Status** | -1.765 (0.26) | 50.49 | <0.001 |
|  | **Pregnancy stage:** |  | 9.50 | 0.009 |
|  | **Mid** | -0.296 (0.32) |  |  |
|  | **Late** | 0.477 (0.29) |  |  |
| Testosterone^a^ | **Status** | -1.190 (0.43) | 6.39 | 0.011 |
|  | **Pregnancy stage:** |  | 9.62 | 0.008 |
|  | **Mid** | 0.819 (0.37) |  |  |
|  | **Late** | 0.963 (0.33) |  |  |
| Log fAm^b,*^ | **Status** | -0.412 (0.15) | 7.60 | 0.006 |
|  | **Pregnancy stage:** |  | 7.19 | 0.027 |
|  | **Late** | 0.145 (0.16) |  |  |
|  | **Postpartum** | -0.212 (0.17) |  |  |
| Results derive from a minimum adequate model. Data are from 52 dams contributing a combined total of 187 samples. All comparisons were made against the indicated levels of each factor: status = dominant; pregnancy stage = early pregnancy (for serum values) or mid pregnancy (for faecal androgen metabolites, fAm); collection period for fAm = AM. χ^2^ = likelihood ratio test statistic (two-tailed test). Bolded terms are significant.  ^a^ GLMM; ^b^ LMM  ^*^ Random effects include litter nested within individual (otherwise, only individual was included in the model) | | | | |

| **Supplementary Table 2 Variables, including status, affecting the behaviour of pregnant and postpartum meerkats** | | | | |
| --- | --- | --- | --- | --- |
| **Behaviour** | **Model terms** | **Estimate (SE)** | **χ^2^** | ***P*** |
| Initiate food competition^a^ | **Status** | -1.916 (0.54) | 12.51 | < 0.001 |
|  | **Clan size** | 0.073 (0.02) | 22.24 | < 0.001 |
| Receive food competition^a^ | **Clan size** | 0.038 (0.02) | 4.92 | 0.026 |
| Initiate intense aggression^b,*^ | **Status** | -1.578 (0.26) | 35.39 | < 0.001 |
|  | **Location** | -0.453 (0.16) | 7.67 | 0.006 |
|  | **Collection period** | -0.319 (0.12) | 7.15 | 0.008 |
|  | **Clan size** | 0.034 (0.01) | 10.25 | 0.001 |
| Receive intense aggression^b,*^ | Status | 0.157 (0.48) | 0.03 | 0.872 |
|  | **Pregnancy stage:** |  | 10.58 | 0.005 |
|  | **Late** | 0.688 (0.30) |  |  |
|  | **Postpartum** | 0.846 (0.32) |  |  |
|  | Location | -0.117 (0.29) | 0.06 | 0.800 |
|  | **Clan size** | 0.035 (0.01) | 5.79 | 0.016 |
|  | **Total monthly rainfall** | -0.012 (0.00) | 8.78 | 0.003 |
|  | **Status*pregnancy stage** |  | 6.30 | 0.043 |
|  | **Status*late** | -1.198 (0.54) |  |  |
|  | **Status*postpartum** | -0.720 (0.66) |  |  |
|  | **Status*location** | 2.235 (0.47) | 22.57 | < 0.001 |
| Initiate prosociality^c,*^ | **Clan size** | 0.030 (0.01) | 3.92 | 0.048 |
| Receive prosociality^c,*^ | **Status** | -0.899 (0.23) | 14.72 | < 0.001 |
|  | Total monthly rainfall | 0.005 (0.00) | 3.23 | 0.072 |
| Scent mark^b,*^ | **Status** | -0.474 (0.21) | 5.31 | 0.021 |
| Results derive from a GLMM that was a minimum adequate model. Data are from 47 pregnancies of 36 unique individuals, observed during 11174 focal sessions. All comparisons were made against the indicated levels of each factor: status = dominant; pregnancy stage = mid pregnancy; location = den; collection period = AM. χ^2^ = likelihood ratio test statistic (two-tailed test); df = 1 for all factors except pregnancy stage, for which df = 2. Bolded terms are significant.  ^a^ Includes focals whilst ranging only; ^b^ includes focals at den and whilst ranging; ^c^ includes focals at den only  ^*^ Random effects include litter nested within individual (otherwise, only litter was included in the model) | | | | |

| **Supplementary Table 3 Effects of maternal condition and offspring age on offspring aggression** | | | | |
| --- | --- | --- | --- | --- |
| **Behaviour** | **Model terms** | **Estimate (SE)** | **χ^2^** | ***P*** |
| Initiate aggression^a,b,*^ | **Mother:** |  | 13.71 | 0.001 |
|  | **Subordinate control** | -0.603 (0.18) |  |  |
|  | **Dominant treated** | -1.121 (0.22) |  |  |
|  | **Offspring age** | -0.352 (0.03) | 264.70 | < 0.001 |
|  | **Group size** | 0.007 (0.01) | 6.66 | 0.010 |
|  | Total monthly rainfall | -0.007 (0.00) | 0.62 | 0.432 |
|  | **Location** | 1.324 (0.09) | 206.97 | < 0.001 |
|  | Collection period | -0.087 (0.05) | 2.89 | 0.089 |
|  | **Mother*offspring age:** |  | 21.64 | < 0.001 |
|  | **Subordinate*offspring age** | 0.063 (0.04) |  |  |
|  | **Dominant treated*offspring age** | 0.197 (0.04) |  |  |
|  | **Group size*total monthly rainfall** | 0.0004 (0.00) | 6.09 | 0.014 |
| Results derive from a GLMM that was a minimum adequate model (MAM). Data are from 103 offspring observed across 5661 focal sessions. All comparisons were made against the indicated levels of each factor: mother = dominant control; location = den; collection period = AM. χ^2^ = likelihood ratio test statistic (two-tailed test); df = 1 for all factors except mother, for which df = 2. Bolded terms are significant.  ^a^ Includes focals at den and whilst ranging; ^b^ offspring sex was not a fixed effect included in the MAM. It was not a significant predictor in the full model and was removed sequentially via AIC.  ^*^ Random effects include individual nested within litter nested within dam (otherwise, only individual nested within litter was included in the model) | | | | |

| **Supplementary Table 4 Effects of antiandrogen-treatment on the behaviour of pregnant and postpartum, dominant meerkats** | | | | |
| --- | --- | --- | --- | --- |
| **Behaviour** | **Model terms** | **Estimate (SE)** | **χ^2^** | ***P*** |
| Initiate food competition^a^ | **Treatment** **condition** | -0.691 (025) | 7.39 | 0.007 |
|  | **Age** | 0.371 (0.06) | 42.18 | < 0.001 |
|  | **Total monthly rainfall** | 0.008 (0.00) | 4.28 | 0.039 |
| Receive food competition^a^ | **Clan size** | 0.038 (0.02) | 5.74 | 0.017 |
| Initiate intense aggression^b,*^ | **Collection period** | -0.304 (0.11) | 7.51 | 0.006 |
|  | Location | -0.295 (0.16) | 3.27 | 0.070 |
|  | **Clan size** | 0.041 (0.01) | 14.07 | < 0.001 |
| Receive intense aggression^b,*^ | Treatment condition | -0.306 (0.18) | 2.83 | 0.092 |
|  | **Clan size** | 0.031 (0.01) | 8.04 | 0.005 |
|  | **Total monthly rainfall** | -0.009 (0.00) | 7.36 | 0.007 |
| Initiate prosociality^c,*^ | Treatment condition | -0.450 (0.29) | 2.46 | 0.117 |
|  | **Clan size** | 0.035 (0.02) | 4.94 | 0.026 |
| Receive prosociality^c^ | **Treatment** **condition** | -0.507 (0.21) | 5.73 | 0.017 |
|  | **Clan size** | 0.022 (0.01) | 5.22 | 0.022 |
| Receive submission^c,*^ | **Treatment** **condition** | -0.902 (0.44) | 4.26 | 0.039 |
|  | **Collection period** | -0.629 (0.29) | 4.81 | 0.028 |
| Scent mark^b,*^ | **Treatment** **condition** | -0.603 (0.23) | 6.94 | 0.008 |
|  | **Location** | 0.630 (0.25) | 6.36 | 0.012 |
|  | **Collection period** | -0.471 (0.15) | 10.34 | 0.001 |
|  | **Clan size** | -0.038 (0.02) | 5.82 | 0.016 |
| Vigilance (rate)^b,*^ | **Treatment** **condition** | -0.142 (0.07) | 4.25 | 0.039 |
|  | **Location** | -0.593 (0.08) | 50.75 | < 0.001 |
|  | **Collection period** | 0.141 (0.04) | 12.86 | < 0.001 |
|  | **Clan size** | -0.021 (0.01) | 11.79 | < 0.001 |
|  | **Total monthly rainfall** | -0.003 (0.00) | 5.94 | 0.015 |
| Vigilance (duration)^b, *^ | **Treatment** **condition** | 0.302 (0.13) | 5.23 | 0.022 |
|  | **Location** | -1.452 (0.11) | 181.80 | < 0.001 |
|  | **Collection period** | -0.505 (0.07) | 47.28 | < 0.001 |
|  | **Clan size** | -0.030 (0.01) | 7.34 | 0.007 |
| Digging (rate)^a,*^ | Collection period | 0.085 (0.05) | 3.24 | 0.072 |
|  | **Total monthly rainfall** | -0.003 (0.00) | 6.46 | 0.011 |
| Digging (duration)^a,*^ | **Collection period** | -0.016 (0.00) | 17.90 | < 0.001 |
| Frequency and duration results derive from a GLMM and LMM, respectively, that were minimum adequate models. Data are from 31 pregnancies of 22 dominant dams, observed during 1044 focal sessions. All comparisons were made against the indicated levels of each factor: treatment condition = control; location = den; collection period = AM. χ^2^ = likelihood ratio test statistic (two-tailed test); df = 1. Bolded terms are significant.  ^a^ Includes focals whilst ranging only; ^b^ includes focals at den and whilst ranging; ^c^ includes focals at den only  ^*^ Random effects include pregnancy stage (late pregnancy and postpartum) and litter nested within individual (otherwise, only pregnancy stage and litter were included in the model) | | | | |

| **Supplementary Table 5 Effects of antiandrogen-treatment of dominant female meerkats on her dyadic rates of nearest-neighbour associations, per adult clan member, as measured by proximity scans** | | | | |
| --- | --- | --- | --- | --- |
| **Behaviour** | **Model terms** | **Estimate (SE)** | ***t* value** | ***P*** |
| Proximity | **Intercept** | 4.427 (0.78) | 5.65 | < 0.001 |
|  | **Treatment condition** | -0.602 (0.20) | -3.04 | 0.004 |
|  | **Clan size** | -0.158 (0.05) | -3.07 | 0.017 |
|  | Partner is a subordinate pregnant female | -0.981 (0.51) | -1.90 | 0.060 |
|  | Partner is male | -0.108 (0.25) | -0.43 | 0.670 |
|  | Partner is the dominant male | 0.563 (0.43) | 1.30 | 0.199 |
| Results derive from LMM using matched control and antiandrogen conditions for four dominant females (i.e., *n* = eight litters in four clans or 110 dyads). *P* values represent two-tailed tests, and bolded terms are significant. Data were obtained from dams in late pregnancy and the early postpartum period, using a scan sampling protocol conducted during focals, both at the den and whilst the animals were foraging. | | | | |

| **Supplementary Table 6 Treatment effects on faecal androgen and glucocorticoid metabolite concentrations and on postpartum weight in dominant meerkat dams** | | | | |
| --- | --- | --- | --- | --- |
| **Variable** | **Model terms** | **Estimate (SE)** | **Test statistic** | ***P*** |
| Log fAm^a,1^ | Treatment condition | -3.712 (0.25) | 2.21 | 0.317 |
| Log fGCm^a,2^ | **Collection period** | 0.785 (0.18) | 19.46 | < 0.001 |
|  | **Pregnancy stage** | -1.418 (0.17) | 66.09 | < 0.001 |
| PP weight^b,3^ | Treatment condition | 3.603 (30.09 | 0.12 | 0.906 |
|  | **Average NP weight** | 0.497 (0.16) | 3.18 | 0.004 |
| Results derive from minimum adequate models (MAM). The MAM for faecal glucocorticoid metabolites (fGCm) did not include treatment condition, which was a non-significant predictor. Comparisons were made against the indicated levels of each factor: pregnancy stage = late pregnancy (for hormone analyses); collection period = AM, treatment condition = control. Other abbreviations: faecal androgen metabolites (fAM); postpartum (PP); not pregnant (NP). Bolded terms are significant.  ^a^ LMM: χ^2^ = likelihood ratio test statistic (two-tailed test); df = 1. ^b^ LM: *t* value = test statistic (two-tailed test); df = 1.  ^1^ Random effects include litter and pregnancy stage; ^2^ random effects include litter identity nested within individual; ^3^ no random effects. | | | | |

| **Supplementary Table 7 Effect of the matriarch’s treatment condition on the behaviour of pregnant and postpartum subordinate meerkats** | | | | |
| --- | --- | --- | --- | --- |
| **Behaviour** | **Model terms** | **Estimate (SE)** | **χ^2^** | ***P*** |
| Initiate food competition^a,*^ | **Matriarch’s treatment** | 1.561 (0.61) | 6.48 | 0.011 |
|  | Collection period | 0.911 (0.59) | 2.41 | 0.120 |
| Receive food competition^a,*^ | **Clan size** | 0.056 (0.02) | 6.06 | 0.014 |
| Initiate intense aggression^b,*^ | **Matriarch’s treatment** | 0.940 (0.37) | 6.55 | 0.010 |
|  | Location | -1.392 (1.04) | 1.79 | 0.181 |
|  | Collection period | 0.524 (0.29) | 3.31 | 0.069 |
| Receive intense aggression^b^ | **Location** | 1.287 (0.40) | 10.57 | 0.001 |
|  | **Clan size** | 0.052 (0.02) | 7.11 | 0.008 |
| Results derive from a GLMM that was a minimum adequate model. Data are from 17 pregnancies of 17 subordinate dams, observed during 388 focal sessions. All comparisons were made against the indicated levels of each factor: matriarch’s treatment = control; location = den; collection period = AM. χ^2^ = likelihood ratio test statistic (two-tailed test); df = 1. Bolded terms are significant.  ^a^ Includes focals whilst ranging only; ^b^ includes focals at den and whilst ranging.  ^*^ Random effects include pregnancy stage (mid pregnancy, late pregnancy, postpartum) and litter (otherwise, only pregnancy stage was included in the model) | | | | |
