# Supplementary material for: An intergenerational androgenic mechanism of female intrasexual competition in the cooperatively breeding meerkat
Source: Nat Commun. 2021 Dec 17;12:7332. doi: 10.1038/s41467-021-27496-x (PMC8683399; doi:10.1038/s41467-021-27496-x)
Supplement: Supplementary file 3 — Reporting Summary [file 41467_2021_27496_MOESM3_ESM.pdf]

nature research

Corresponding author(s): Christine Drea

Last updated by author(s): Oct 20, 2021

# Reporting Summary

Nature Research wishes to improve the reproducibility of the work that we publish. This form provides structure for consistency and transparency in reporting. For further information on Nature Research policies, see our Editorial Policies and the Editorial Policy Checklist.

## Statistics

For all statistical analyses, confirm that the following items are present in the figure legend, table legend, main text, or Methods section.

| n/a | Confirmed | |
|---|---|---|
| ☐ | ☒ | The exact sample size (*n*) for each experimental group/condition, given as a discrete number and unit of measurement |
| ☐ | ☒ | A statement on whether measurements were taken from distinct samples or whether the same sample was measured repeatedly |
| ☐ | ☒ | The statistical test(s) used AND whether they are one- or two-sided<br>*Only common tests should be described solely by name; describe more complex techniques in the Methods section.* |
| ☐ | ☒ | A description of all covariates tested |
| ☐ | ☒ | A description of any assumptions or corrections, such as tests of normality and adjustment for multiple comparisons |
| ☐ | ☒ | A full description of the statistical parameters including central tendency (e.g. means) or other basic estimates (e.g. regression coefficient) AND variation (e.g. standard deviation) or associated estimates of uncertainty (e.g. confidence intervals) |
| ☐ | ☒ | For null hypothesis testing, the test statistic (e.g. *F*, *t*, *r*) with confidence intervals, effect sizes, degrees of freedom and *P* value noted<br>*Give P values as exact values whenever suitable.* |
| ☒ | ☐ | For Bayesian analysis, information on the choice of priors and Markov chain Monte Carlo settings |
| ☒ | ☐ | For hierarchical and complex designs, identification of the appropriate level for tests and full reporting of outcomes |
| ☐ | ☒ | Estimates of effect sizes (e.g. Cohen's *d*, Pearson's *r*), indicating how they were calculated |

*Our web collection on statistics for biologists contains articles on many of the points above.*

## Software and code

Policy information about availability of computer code

| | |
|---|---|
| Data collection | No software was used to collect endocrine data. Behavioural data were collected using CyberTracker software (version 3.263, CyberTracker Conservation). |
| Data analysis | All of the data were analysed in the R software environment, as detailed below.<br>Endocrine analyses: R (version 3.4.3); GLMMs in the MASS package (version 7.3-47); Gamma error distribution and log link function; LMMs in the lme4 package (version 1.1-21); Gaussian error distribution with an identity link function; variance inflation factors (VIFs) in R package “car” (version 2.1-6); maximum likelihood estimation and likelihood ratio tests following a χ2 distribution; post hoc pairwise comparisons (LSD) in the lsmeans package (version 2.30-0).<br>Adult behavioural analyses: zero-inflated GLMMs in the glmmADMB package (version 0.8.3.3) in R (version 3.4.3); Poisson or negative binomial error distribution, with Akaike’s Information Criterion (AIC) value; maximum likelihood estimation and likelihood ratio tests following a χ2 distribution; post hoc pairwise comparisons (LSD) in the lsmeans package (version 2.30-0)<br>Nearest-neighbour analyses: LMM in R (version 3.6.1), using the lme4 package (version 1.1-21); VIFs using the R package “car” (version 3.0-5).<br>Offspring behaviour: zero-inflated GLMMs in the glmmTMB package (version 1.0.1) in R (version 3.6.3); negative binomial error distribution; AIC, with model confirmed using a forward stepwise procedure; Fixed factor significance determined through maximum likelihood estimation and likelihood ratio tests following a χ2 distribution; post hoc pairwise comparisons (LSD) in the lsmeans package (version 2.30-0). |

For manuscripts utilizing custom algorithms or software that are central to the research but not yet described in published literature, software must be made available to editors and reviewers. We strongly encourage code deposition in a community repository (e.g. GitHub). See the Nature Research guidelines for submitting code & software for further information.

## Data

Policy information about availability of data

All manuscripts must include a data availability statement. This statement should provide the following information, where applicable:

- Accession codes, unique identifiers, or web links for publicly available datasets
- A list of figures that have associated raw data
- A description of any restrictions on data availability

These data are available at https://github.com/cls83211/dreaetal2021

# Field-specific reporting

Please select the one below that is the best fit for your research. If you are not sure, read the appropriate sections before making your selection.

☒ Life sciences ☐ Behavioural & social sciences ☐ Ecological, evolutionary & environmental sciences

For a reference copy of the document with all sections, see nature.com/documents/nr-reporting-summary-flat.pdf

# Life sciences study design

All studies must disclose on these points even when the disclosure is negative.

| | |
|---|---|
| Sample size | Target samples sizes were estimated/calculated for the NSF grant that supported this study. Ultimately, final sample sizes were determined by animal/clan availability (i.e., there is only 1 dominant female per clan), by the degree of synchrony between the pregnancies of dominant and subordinate females, by the litter sizes they produced, and by animal survival under natural conditions (e.g. barring abortions, predation, etc.). |
| Data exclusions | There were no preestablished exclusion criteria. Data were excluded if sample sizes achieved were insufficient to accurately perform the statistical analysis. Accordingly, there was no analysis of normative status differences in faecal androgen metabolites during early pregnancy (Fig 1c), because too few faecal samples were obtained during this pregnancy stage (Table 1). Likewise, there were too few occurrences of evictions by dominant treated dams to allow a robust analysis of treatment effects on evictions (Fig 3e). Data were excluded from focals during which there was an intergroup encounter and data fwere excluded from pregnant females that subsequently aborted. |
| Replication | This study involved 1 experiment that spanned five years - there was no replication. The measures taken to verify reproducibility involved (1) obtaining the largest sample sizes of dominant control, subordinate control, and dominant treated dams, and their respective pups, as possible, and (2) addressing multiple predictions of the same hypothesis, all of which led to the same interpretation. |
| Randomization | Candidate dominant females for treatment were identified by a project veterinarian based on their health status, our confidence in their pregnancy estimation, and the current stability/size/health of their clan. Once identified, they were randomly assigned to control or treatment groups. Otherwise, randomization was not relevant to this study: Subordinate dams were 'self' identified based on whether or not they became pregnant concurrently; offspring were automatically 'assigned' according to their mother's treatment or status, and their survivorship over time. |
| Blinding | Given the long-term, large-scale nature of this study, the researchers (i.e., the veterinarians and successive project managers) who performed or monitored the treatments were different from those who successively performed the bulk of the behavioral observations. With few exceptions, personnel who performed the data and sample collection in Africa differed from the personnel who performed final data curation, laboratory analyses, or statistical analyses in the US. |

# Reporting for specific materials, systems and methods

We require information from authors about some types of materials, experimental systems and methods used in many studies. Here, indicate whether each material, system or method listed is relevant to your study. If you are not sure if a list item applies to your research, read the appropriate section before selecting a response.

## Materials & experimental systems

| n/a | Involved in the study | |
|---|---|---|
| ☒ | ☐ | Antibodies |
| ☒ | ☐ | Eukaryotic cell lines |
| ☒ | ☐ | Palaeontology and archaeology |
| ☐ | ☒ | Animals and other organisms |
| ☒ | ☐ | Human research participants |
| ☒ | ☐ | Clinical data |
| ☒ | ☐ | Dual use research of concern |

## Methods

| n/a | Involved in the study | |
|---|---|---|
| ☒ | ☐ | ChIP-seq |
| ☒ | ☐ | Flow cytometry |
| ☒ | ☐ | MRI-based neuroimaging |

## Animals and other organisms

Policy information about studies involving animals; ARRIVE guidelines recommended for reporting animal research

| | |
|---|---|
| Laboratory animals | The study did not involve laboratory animals. |
| Wild animals | This study was conducted on wild meerkats (Suricata suricatta) over a period of 5 years, with an annual mean population size of 270 animals. Our focal subjects included 57 adult dams of varying ages (Tables 1 and 2) and their 103 offspring, including 47 females, 55 males, and 1 infant of unidentified sex, followed from birth to maximally 6 mo of age (Table 3). As the animals were habituated to humans, captures involved walking up to a target individual, gently picking them up by the base of the tail, placing them into a cotton sack, and walking them over to a nearby vehicle, equipped for on-site processing (there was no other transport). Captures were temporary (for treatment or sampling alone) and, after recovery from anaesthesia, all captured animals were released back to their groups within about 30 min. No animals were euthanized as part of this study. |
| Field-collected samples | All of the samples (blood and faeces) were collected in the field (in South Africa) from wild animals. The samples were minimally processed on site, stored frozen, and then transported to the US, on ice, and fully processed and analysed in the Drea laboratory at Duke University. None of the samples required any animal housing. |
| Ethics oversight | Our protocols were approved by and carried out in accordance with the Institutional Animal Care and Use Committee of Duke University (Protocol Registry Numbers A171-09-06 and A143-12-05) and the University of Pretoria's Animal Use and Care Committee (Ethical Approval Number EC074-11). |

Note that full information on the approval of the study protocol must also be provided in the manuscript.
